# Synergistic Interaction Between *Justicia spicigera* Extract and Analgesics on the Formalin Test in Rats

**DOI:** 10.3390/ph18020187

**Published:** 2025-01-30

**Authors:** Juan Ramón Zapata-Morales, Angel Josabad Alonso-Castro, María Leonor González-Rivera, Hugo Israel González Prado, Juan Carlos Barragán-Gálvez, Araceli Hernández-Flores, María del Carmen Juárez-Vázquez, Fabiola Domínguez, Candy Carranza-Álvarez, Amaury de Jesús Pozos-Guillén, Juan F. López-Rodríguez, Patricia Aguirre-Bañuelos, Marco Antonio Ramírez-Morales

**Affiliations:** 1 Department of Pharmacy, Natural and Exact Sciences Division, University of Guanajuato (UG), Guanajuato 36050, Mexico; angeljosabad@ugto.mx (A.J.A.-C.); leonor.glez.rivera@outlook.com (M.L.G.-R.); hi.gonzalezprado@ugto.mx (H.I.G.P.); jcbarragang@gmail.com (J.C.B.-G.); aracelihf@hotmail.com (A.H.-F.); carmenjuarezv@gmail.com (M.d.C.J.-V.); marco.ramirezmo@hotmail.com (M.A.R.-M.); 2 Eastern Biomedical Research Center of Mexican Social Security Institute (IMSS), Metepec 74360, Mexico; irma.dominguez@imss.gob.mx; 3 School of Professional Studies Huasteca Zone, Autonomous University of San Luis Potosí, Ciudad Valles 79060, Mexico; candy.carranza@uaslp.mx; 4 Basics Sciences Laboratory, School of Dentistry, Autonomous University of San Luis Potosi (UASLP), San Luis Potosí 78290, Mexico; apozos@uaslp.mx; 5 Animal Laboratory, School of Medicine Autonomous University of San Luis Potosi, San Luis Potosi 78290, Mexico; francisco.lopez@uaslp.mx; 6 Faculty of Chemical Sciences, Autonomous University of San Luis Potosí, Manuel Nava Martínez 6 Avenue, San Luis Potosí 78210, Mexico; paguirreb@uaslp.mx

**Keywords:** *Justicia spicigera*, naproxen, tramadol, formalin test, isobolographic analysis, synergism

## Abstract

**Background:** Combining antinociceptive drugs with different mechanisms of action can reduce the doses and the adverse effects, with a possible increase in the antinociceptive effect. This work evaluated the antinociceptive effect of the combination of an ethanol extract of *Justicia spicigera* (JSE) with naproxen (NPX) or tramadol (TML) using the formalin test in rats. **Methods:** Rats received JSE (30–200 mg/kg p.o.), NPX (50–300 mg/kg p.o.), or TML (5–50 mg/kg p.o.) 60 min before paw administration with formalin (5%). Different proportions of the combination between NPX and JSE, as well as TML and JSE, were used in the formalin test to obtain the dose–response curve of each drug and the experimental effective dose 50 (ED_50_). The levels of IL-1β and COX2 were assessed using a Western blot analysis as a possible mechanism of action for the combination of JSE and analgesics. A pharmacokinetic study was conducted to evaluate the effect of JSE on the pharmacokinetic parameters of NPX. **Results:** The ED_50_ values for the proportions NPX:JSE were 107.09 mg/kg (1:1), 102.44 mg/kg (3:1), and 73.82 mg/kg (1:3). The ED_50_ values for the proportions TML:JSE were 66 mg/kg (1:1), 29.5 mg/kg (1:3), and 78 mg/kg (3:1). The combination NPX:JSE (1:3) showed the best synergistic interaction index (0.501). The pharmacokinetic study revealed that there were no significant changes in the pharmacokinetic parameters of NPX administered individually and the combination NPX:JSE. **Conclusions:** In this preclinical study, the combination NPX:JSE showed antinociceptive effects by decreasing the levels of COX2 and IL-1β without affecting NPX’s pharmacokinetics.

## 1. Introduction

Since pre-Hispanic times, Mexican traditional medicine has used *Justicia spicigera*, also known as “muitle” or “muicle”, as an empirical treatment for pain, superficial wounds or injuries, fever, inflammation, and liver infections [1,2].

Numerous studies have demonstrated the in vitro and in vivo effectiveness of *Justicia spicigera* in treating anemia and cell damage [3,4], infections and parasites [5], high blood pressure [4], and inflammation [6,7]; boosting the immune system; and fighting tumors [8]. In addition, Zapata-Morales et al. [9] showed that an ethanol extract from *Justicia spicigera* leaves (JSE) exerted antinociceptive activity in the acetic acid writhing test, the formalin test in both phases, and the tail-flick test. The antinociceptive effects of JSE (200 mg/kg p.o.) were like those of naproxen (150 mg/kg p.o.) in the acetic acid writhing test and phase 2 of the formalin test.

The treatment of pain involves the use of different drugs, such as non-steroidal anti-inflammatory analgesics (NSAIDs) and opioids, among others, according to the degree and location of pain [10]. Another alternative includes a combination of two or more types of treatments. Combining two painkillers with different mechanisms of action could potentially address the issues related to their effectiveness, duration of action, and tolerance. In some cases, it might even make the analgesics work better together [11]. Combining analgesics with different types of activities can reduce the doses of the drugs, leading to a reduction in the number of prescribed drugs, a decrease in the possibility of adverse effects, and potentially an increase in the pharmacological effect. A combination of drugs may extend the duration of the therapeutic effect without exacerbating the adverse effects compared to individual drug administration.

No study has examined the combination of *Justicia spicigera* with NSAIDs or opioids. Therefore, the present study aimed to evaluate the pharmacodynamic interaction between the ethanolic extract of *Justicia spicigera* and naproxen or tramadol, two of the most widely used analgesics in the world. The molecular mechanism of the antinociceptive effect of the combination of JSE and naproxen was evaluated. In addition, this study assessed the effect of JSE on the pharmacokinetics of naproxen.

## 2. Results

### 2.1. Kaempferitrin (KM) Quantitation

**Figure 1:**
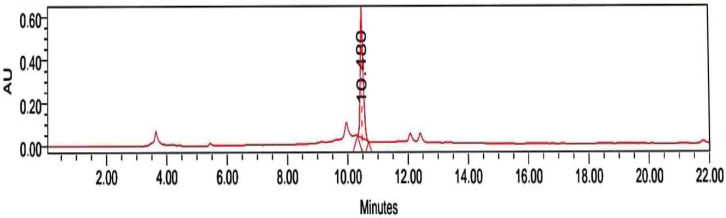
Chromatogram of the Justicia spicigera extract (JSE) showing the detection of kaempferitrin.

KM is the active compound in JSE. The retention time of KM was 10.48 min, and the content of KM in JSE was 42 mg/g (Figure 1).

### 2.2. Antinociceptive Activity of JSE, NPX, and TML

**Figure 2:**
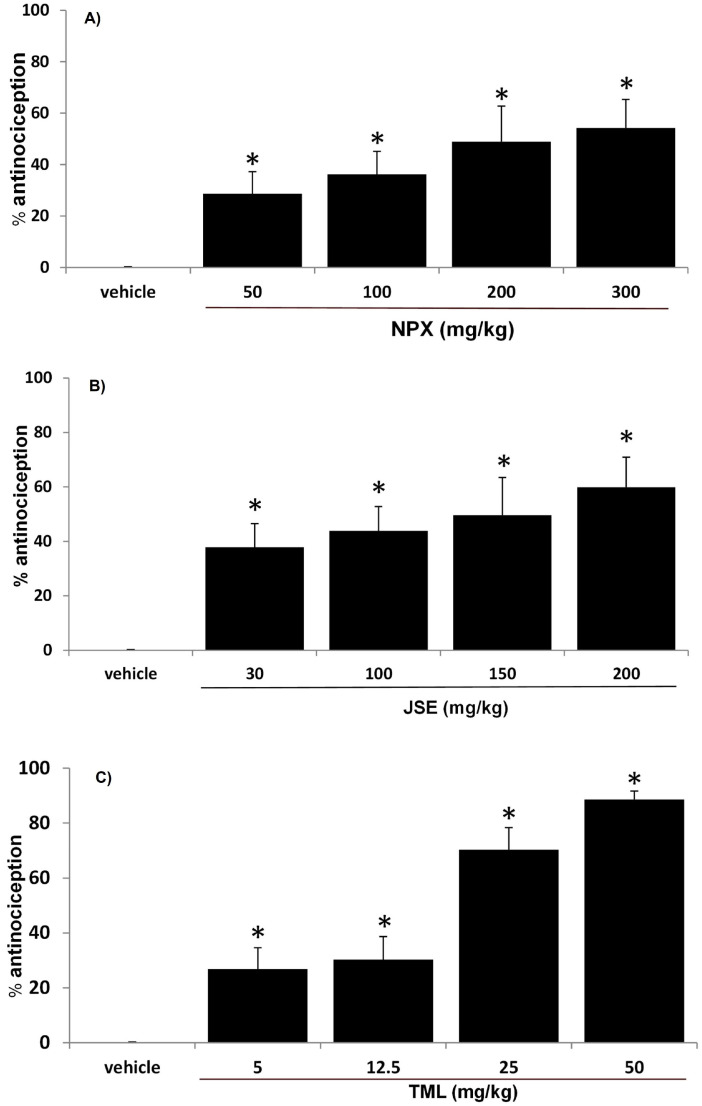
The formalin test in the rats demonstrated the antinociceptive effect of naproxen (**A**), the *Justicia spicigera* extract (**B**), and tramadol (**C**). The vertical bars represent the mean ± S.E.M. for each experimental group (n = 6). * *p* < 0.05 versus vehicle group. ANOVA followed by Dunnett’s test.

JSE, NPX, and TML showed a dose-dependent antinociceptive activity (Figure 2). The highest doses tested, 200 mg/kg JSE, 300 mg/kg NPX, and 50 mg/kg TML, exhibited antinociceptive activities of 59.87%, 54.29%, and 89%, respectively (Figure 2). The ED_50_ values were 121.15 mg/kg (JSE), 226.21 mg/kg (NPX), and 14.58 mg/kg (TML).

### 2.3. Antinociceptive Activity of JSE, NPX, and TML

**Figure 3:**
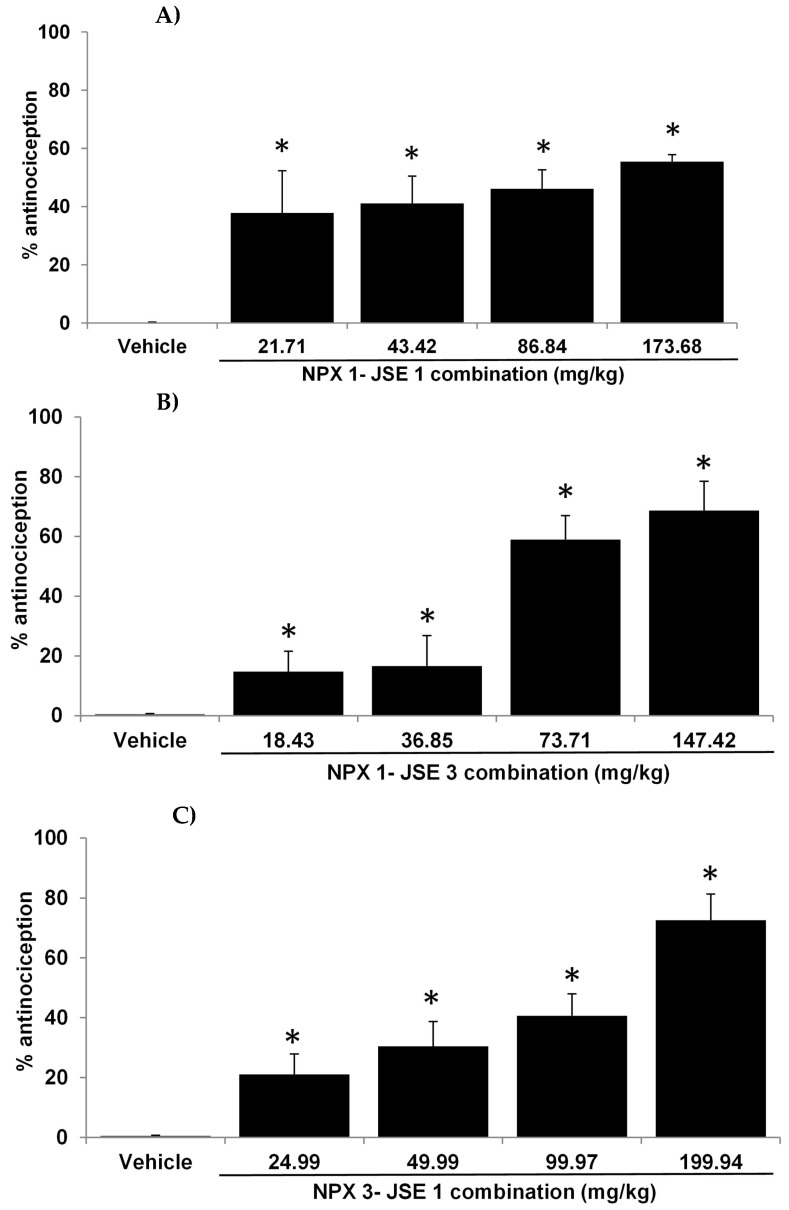
The formalin test demonstrated the antinociceptive effect of the Naproxen (NPX)–Justicia spicigera extract (JSE) combination at ratios of (**A**) 1:1 (NPX 113.1 mg/k +60.58 mg/kg = 173.68 mg/kg), (**B**) 1:3 (NPX 56.6 mg/kg + 90.9 mg/kg = 147.42 mg/kg), and (**C**) 3:1 (NPX 169.7 mg/kg + 30.3 mg/kg = 199.94 mg/kg). The vertical bars represent the mean ± S.E.M. for each experimental group (n = 6). * *p* < 0.05 versus the vehicle group. ANOVA followed by the Dunnett’s test.

The combination NPX-JSE and its three proportions (1:1, 1:3, and 3:1) showed antinociception in a dose-dependent manner (Figure 3). The highest values of antinociception were recorded as 55.44% (173.68 mg/kg p.o. 1:1 NPX-JSE), 68.67% (147.42 mg/kg p.o. 1:3 NPX-JSE), and 72.54% (199.94 mg/kg p.o. 3:1 NPX-JSE). The ED_50_ values for the proportions NPX:JSE were 107.09 mg/kg (1:1), 73.82 mg/kg (1:3), and 102.44 mg/kg (3:1).

**Figure 4:**
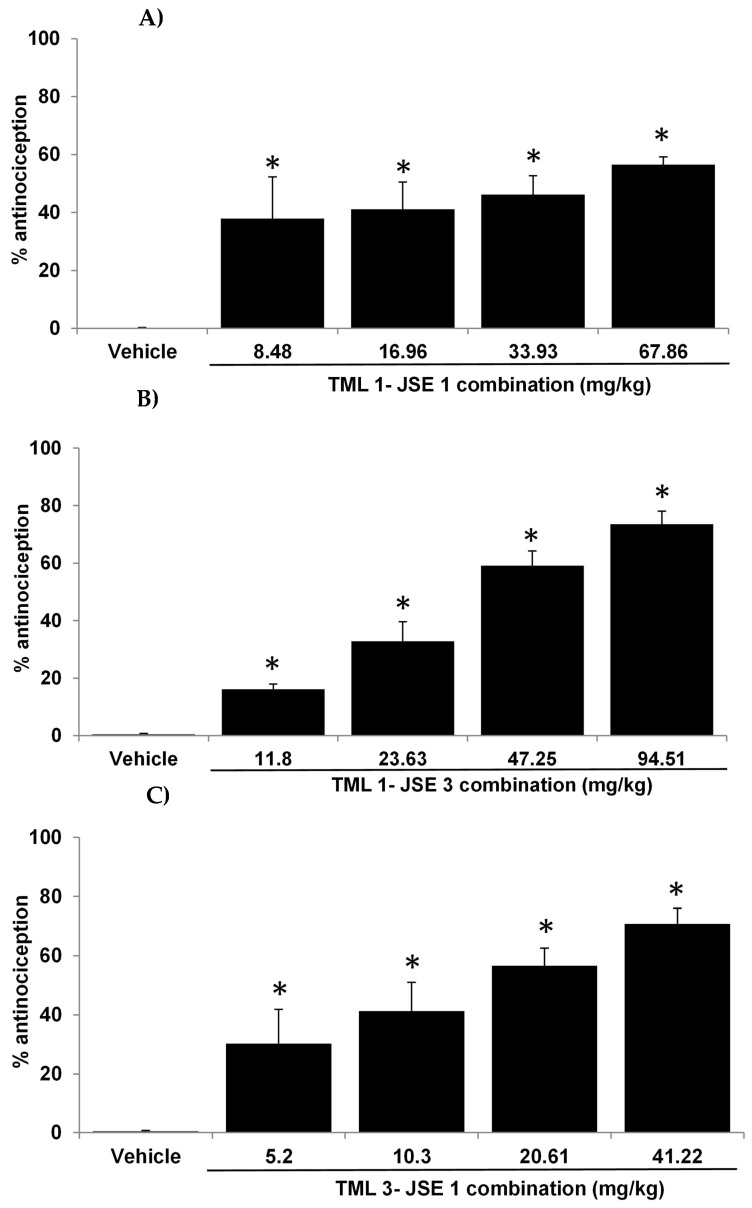
Antinociceptive effect of the tramadol (TML)–Justicia spicigera extract (JSE) combination: (**A**) 1:1 (TML 7.29 mg/kg + 60.58 mg/kg = 67.86 mg/kg), (**B**) 1:3 (TML 3.6 mg/kg + 90.9 mg/kg = 94.51 mg/kg), and (**C**) 3:1 (TML 10.9 mg/kg + 30.3 mg/kg = 41.22 mg/kg) ratios in the formalin test. The vertical bars represent the mean ± S.E.M. for each experimental group (n = 6). * *p* < 0.05 versus the vehicle group. ANOVA followed by the Dunnett’s test.

The combination of TML-JSE and its proportions exhibited dose-dependent antinociceptive activity (Figure 4). The highest doses registered the maximum antinociceptive effect as follows: 51.68% (67.86 mg/kg p.o. 1:1 TML-JSE), 73.54% (94.51 mg/kg p.o. 1:3 TML-JSE), and 70.79% (41.22 mg/kg p.o. 3:1 TML-JSE) (Figure 4). The ED_50_ values for the proportions TML:JSE were 66 mg/kg (1:1), 39.23 mg/kg (1:3), and 14.79 mg/kg (3:1). The proportions 3:1 (NPX-JSE) (Figure 3) and 1:3 (TML:JSE) (Figure 4) registered the highest antinociceptive effects.

### 2.4. Isobolographic Analysis

**Figure 5:**
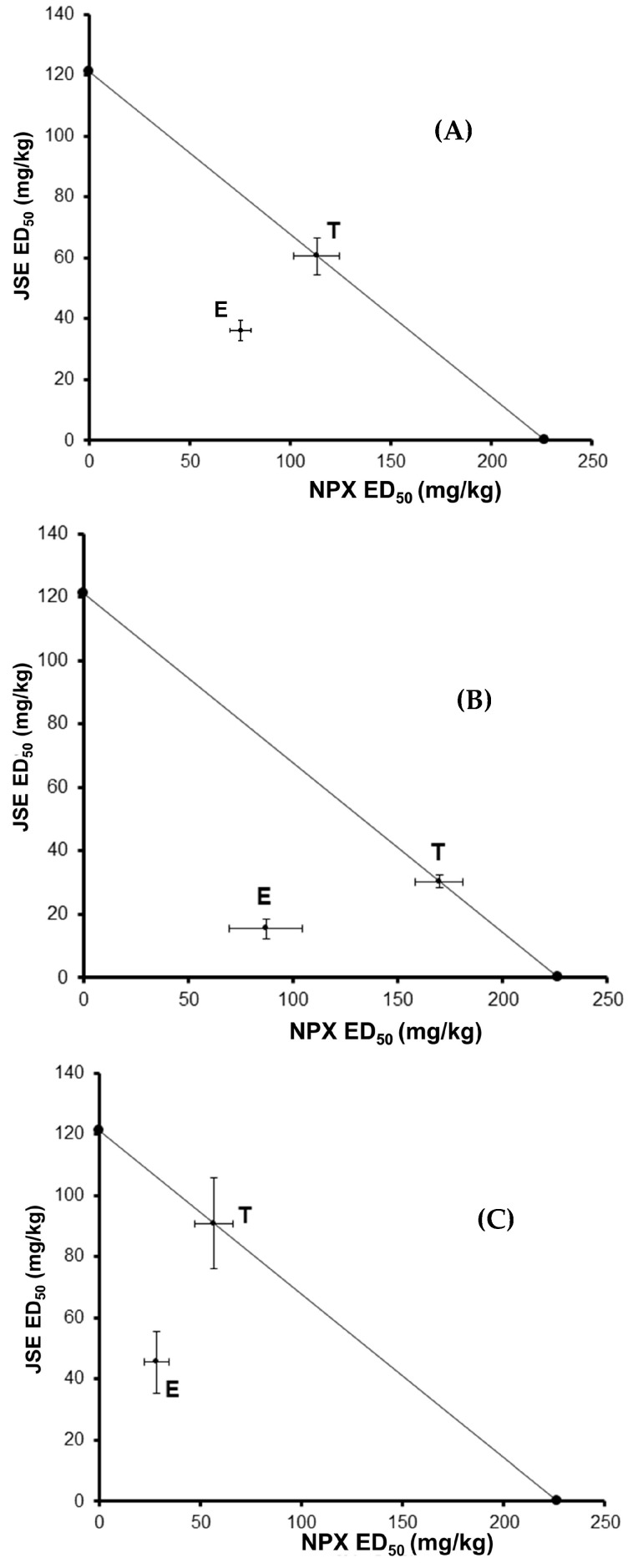
The isobolograms illustrate the experimental interaction between naproxen (NPX) and the Justicia spicigera extract (JSE) at the following ratios: (**A**) 1:1, (**B**) 3:1, and (**C**) 1:3 in the formalin test. T: theoretical additive ED_50_; and E: experimental ED_50_. The points represent the mean ± S.E.M. for each experimental group (n = 6).

**Figure 6:**
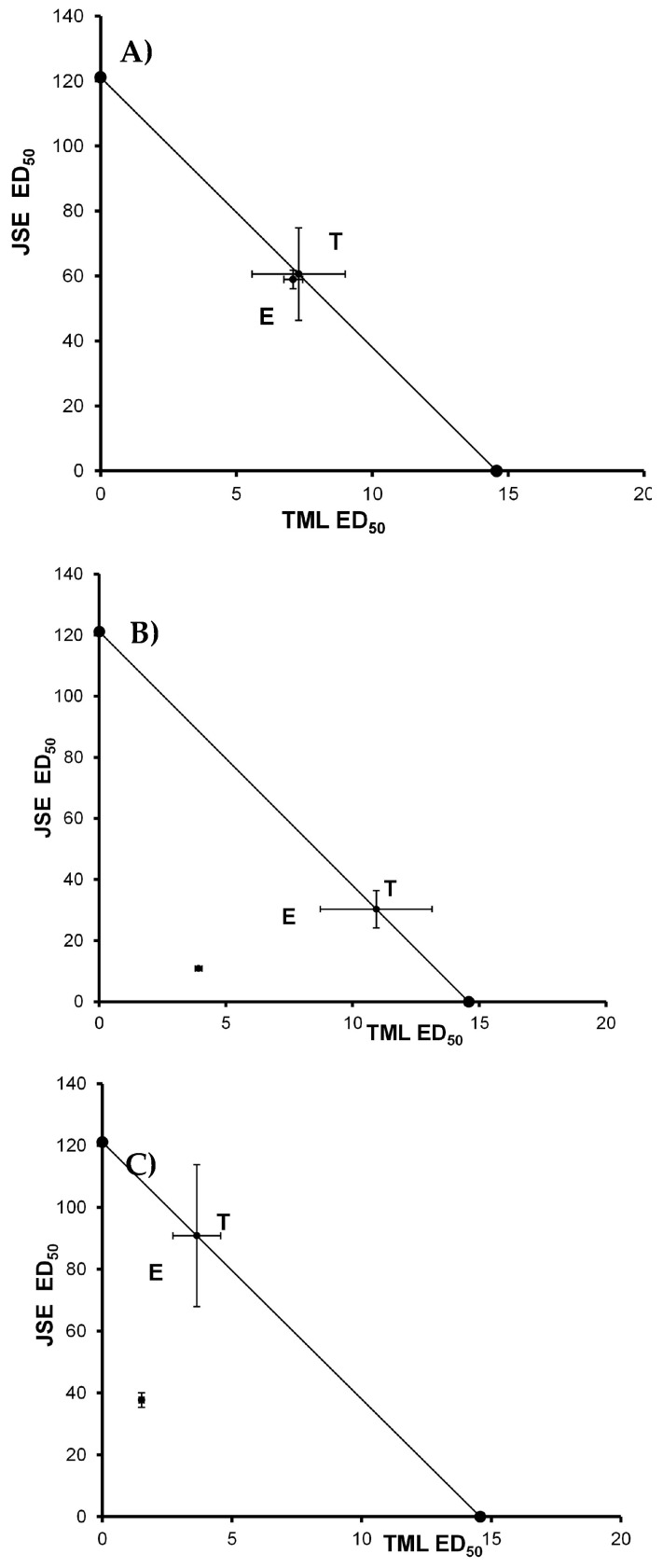
The isobolograms illustrate the experimental interaction between tramadol (TML) and the Justicia spicigera extract (JSE) at the following ratios: (**A**) 1:1, (**B**) 3:1, and (**C**) 1:3 in the formalin test. T: theoretical additive ED_50_; and E: experimental ED_50_. The points represent the mean ± S.E.M. for each experimental group (n = 6).

In the isobolographic analysis, the combination of NPX-JSE and its three proportions demonstrated a synergistic type of interaction, with the antinociceptive effect graphically placed below the line of additivity (Figure 5). The values of γ were 0.617 (1:1 NPX:JSE), 0.512 (3:1 NPX:JSE), and 0.501 (1:3 NPX:JSE) (Table 1). The values of the interaction index were below one, representing a synergistic effect in the pharmacological combination NPX:JSE (Table 1). Similarly, the combination TML:JSE and its three proportions showed synergism (Figure 6). The values of γ were 0.973 (1:1 TML:JSE), 0.359 (3:1 TML:JSE), and 0.415 (1:3 TML:JSE) (Table 2).

### 2.5. Western Blot Analysis

**Figure 7:**
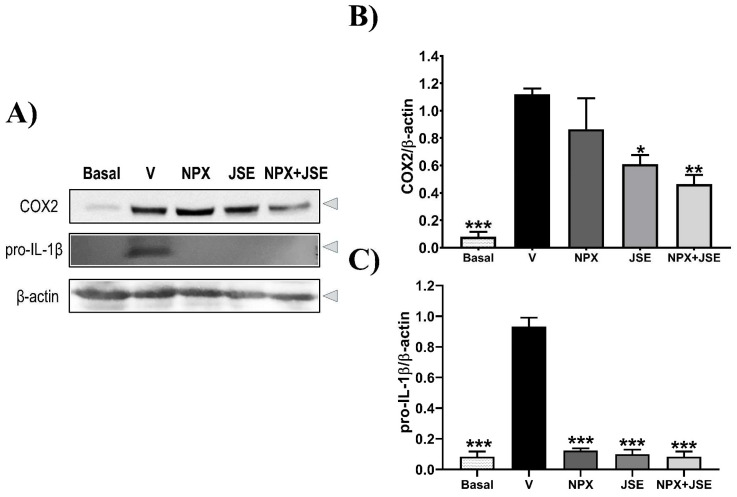
Expression of COX2 and pro-IL-1β in the formalin-induced pain model. As shown in Figure (**A**), COX2 and pro-IL-1β levels were measured in tissues from the right hind paw that had been treated with a vehicle (V), naproxen (NPX), and naproxen mixed with the *Justicia spicigera* extracts (NPX + JSE) ED_50_ = 73.82 mg/kg. The left hind paw not treated with formalin and drugs is denoted with basal, and the saline solution was the vehicle. The COX2 (**B**) and pro-IL-1β (**C**) protein expression underwent a densitometry analysis, n = 6. The data are presented as the mean ± standard error of the mean (S.E.M) ((*p < 0.05; **p < 0.01; *** *p* < 0.001 compared to the vehicle group ).

The combination of 1:3 NPX:JSE demonstrated the best synergistic effect, exhibited a low ED_50_ value of 73.82 mg/kg, and accounted for the use of NSAID. Therefore, this work conducted further experiments with this combination to evaluate its molecular mechanism of action. The expression of COX2 was detected in the vehicle group, and it was slightly decreased in the NPX group (Figure 7A, Lane 2–3 from the upper blot). JSE decreased (*p* < 0.05) the COX2 expression in the rats using the formalin test (Figure 7B). However, the combination NPX:JSE showed a higher reduction (*p* < 0.05) in COX2 expression compared to JSE alone (Figure 7B). Only the group receiving a saline solution exhibited the pro-inflammatory cytokine IL-1. The groups treated with NPX, JSE, or the combination NPX:JSE did not exhibit this cytokine (Figure 7A,C).

### 2.6. Myeloperoxidase (MPO) Activity

**Figure 8:**
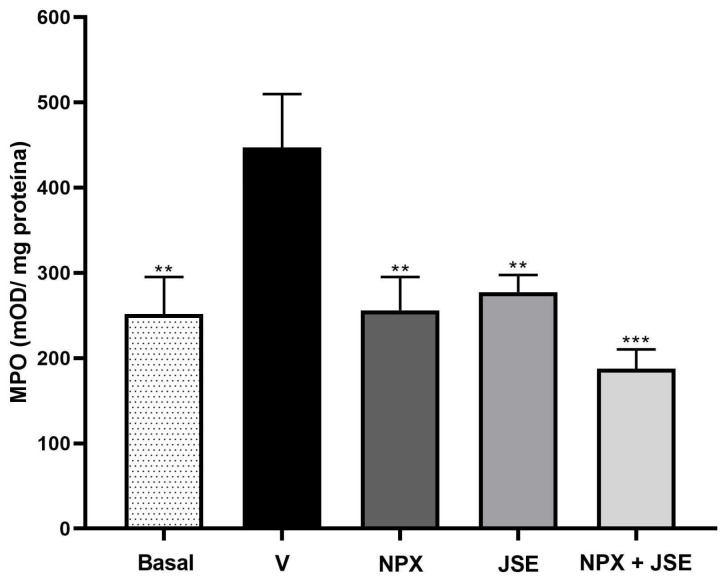
Myeloperoxidase activity in the formalin-induced pain model. The right hind paw was pretreated with the vehicle (V), naproxen (NPX), and naproxen with the *Justicia spicigera* extract (NPX + JSE) plus formalin. The left hind paw was not treated with formalin (basal). The bars represent the mean values (±SEM). n = 6, ** *p* < 0.01, and *** *p* < 0.0001 compared to the vehicle group.

The effect of the combination of 1:3 NPX:JSE on myeloperoxidase activity was evaluated. The activity of MPO in the vehicle group was higher than the left paw or basal without formalin (*p* < 0.01). The right paws of the rats pretreated with formalin—NPX, JSE, or a combination of NPX and JSE—decreased the MPO activity (*p* < 0.05) by 42.73, 37.94, and 58%, respectively. This effect was comparable to the MPO levels in the left paw (Figure 8).

### 2.7. Effect of JSE on the Pharmacokinetics of NPX

**Figure 9:**
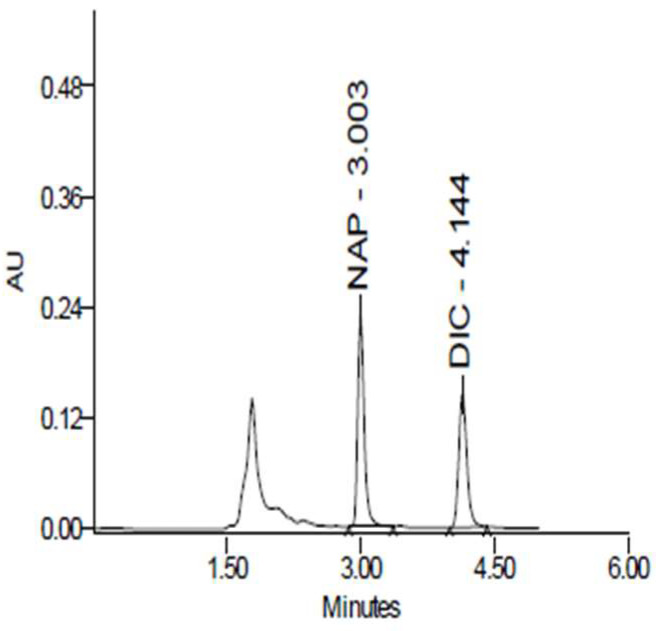
Chromatogram showing the retention times for NPX (3.007 min) and dicloxacillin (4.1 min).

**Figure 10:**
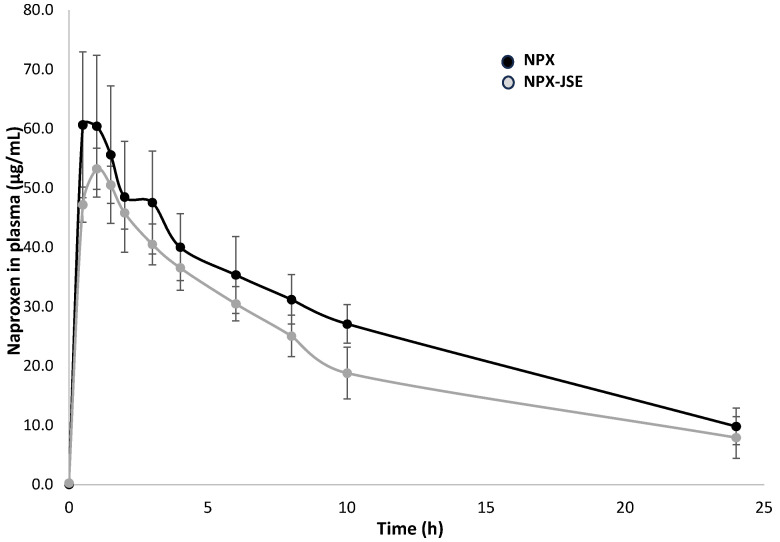
Mean arterial plasma concentration–time profiles of naproxen (NPX) after oral administration at a dose of 30 mg/kg (●; n = 6) or naproxen at a dose of 30 mg/kg with JSE at a dose of 60 mg/kg (○; n = 6). The bars represent standard deviations.

Figure 9 indicates the chromatogram showing the retention times for NPX (3.007 min) and dicloxacillin (4.1 min). Figure 10 depicts the mean arterial plasma concentration–time profiles of naproxen after its oral administration to the rats with or without the simultaneous oral administration of JSE. Table 3 presents the main pharmacokinetic (PK) parameters of naproxen when administered alone or with JSE, along with the statistical test results. As shown in Table 3, after oral administration, NPX was absorbed and reached a maximum concentration of 68.6  ±  11.73 µg/mL at 1.25 h. The combination of JSE and NPX reached a maximum concentration of 55.15  ±  2.39 µg/mL at 1.17 h. There were no significant changes in the pharmacokinetic parameters of NPX administered individually and the combination NPX:JSE.

## 3. Discussion

Balanced analgesia proposes a new pharmacological alternative for the treatment of pain. Drugs such as naproxen have been combined with other analgesics, i.e., paracetamol, to promote changes to individual treatment and provide various benefits, mainly at the therapeutic level [11].

Previous studies of *Justicia spicigera* have found the presence of kaempferol, kaempferol-3, 7-bisrhamnósido (kaempferitrin), cryptoxanthin, allantoin, and β-glucosyl-o-sitosterol [12,13]. This work found kaempferitrin content similar to the previous study [9]. An ethanol extract of *Justicia spicigera* exerted antinociceptive activity in different murine models [9]. However, the antinociceptive mechanism of *Justicia spicigera* remains undefined, underscoring the importance of evaluating its mechanism of action and its combination with analgesics in future studies.

This study used the formalin test, a pain model that involves two phases of pain: the first phase corresponds to neurogenic pain, which includes substance P and bradykinin [14,15], and the second phase corresponds to inflammatory pain, which is characterized by the appearance of inflammation mediators like histamine, serotonin, prostaglandins, and even bradykinin itself [16,17]. The individual administration of JSE, NPX, and TML showed antinociceptive activity in a dose-dependent manner. The antinociceptive effect of these treatments was observed in the following order: TML > JSE > NPX.

The combination of JSE with NPX and TML in three different proportions (1:1, 3:1, and 3:1) showed an increase in antinociception in the formalin test. The combination also demonstrated the existence of synergism between both treatments when combined, confirmed by the interaction index of less than one and in the isobolographic analysis. The proportions 3:1 (NPX-JSE) and 1:3 (TML:JSE) registered the highest antinociceptive effects, whereas the combination 3:1 (NPX-JSE) showed the highest synergism. Further studies were carried out with this combination. Synergism occurs because the drugs’ mechanisms of action differ, causing antinociception. It is known that naproxen works by blocking cyclooxygenase (COX-1 and COX-2) [18]. This stops the production of prostaglandins from arachidonic acid, which is directly connected to controlling pain. Tramadol’s antinociceptive activity stems from its affinity for the opioid and the neuropeptide substance P receptor, as well as the participation of K+ channels and the nitric oxide synthase [19,20].

Canonically, pro-IL-1β is converted to the mature IL-1β form mediated by caspase-1 cleavage after inflammasome formation [21]. IL-1β has been described as capable of inducing COX2 expression through IL-1β receptors and protein kinase C (PKC) signaling [22]. COX2, for its part, induces the production of prostaglandin E2 (PGE2), which plays a role in the generation of inflammatory and neuropathic pain [23]. Myeloperoxidase, an enzyme present in primary azurophilic granules of neutrophils, is another inflammatory mediator. MPO catalyzes the formation of reactive oxygen intermediates such as hypochlorous (HOCl), hypobromous, and hypothiocyanous acids [24]. This work detected the expression of pro-IL-1β (~30 kDa) in the vehicle group where there is inflammation and pain, and the levels of this cytokine were reduced with the treatment of NPX, JSE, and the combination NPX:JSE. The levels of COX2 (~70 kDa) decreased with the treatment of NPX and JSE and markedly reduced with the combination NPX:JSE. Additionally, the MPO activity was lower in the treated groups with NPX, JSE, and the combination NPX:JSE compared to the vehicle group (formalin without treatment). This study found that JSE reduces pain by lowering the levels of COX-2 and IL-1β and the MPO activity in the formalin test in rats. The combination NPX:JSE exerts its antinociceptive actions by markedly decreasing the MPO activity and the COX2 levels, and reducing the IL-1β levels.

In this study, KM was the main compound found in JSE. The main compound in a rich flavonoid extract of lotus (*Nelumbo nucifera*) leaves was KM. The mice that received this plant extract daily for 14 days at a dose of 100 mg/kg p.o. experienced a 74% reduction in ethanol-induced stomach damage. This plant extract increased the levels of nitric oxide and IFN-gamma and lowered the levels of IL-6, IL-12, and TNF-alpha in gastric tissue [25]. These results indicated that KM is participating in the gastroprotective actions of lotus extract. Therefore, KM is unlikely to produce gastric damage in rodents.

The pharmacokinetic parameters of NPX found in this work agree with previous reports in rats [26]. CYP2A1 and CYP2C9 enzymes metabolize NPX [27,28]. JSE did not affect the pharmacokinetic parameters of NPX. Therefore, JSE did not affect the bioavailability (absorption, distribution, or elimination) of NPX. NSAIDs like naproxen present the risk of inducing bleeding. The coadministration of JSE and NPX decreases the possibility of this side effect.

## 4. Materials and Methods

### 4.1. Reagents and Drugs

Tris, sodium chloride, Tween-20, non-fat dry milk, sodium dodecyl sulfate (SDS), naproxen sodium (NPX), tramadol (TML), and acrylamide were from Sigma-Aldrich (St. Louis, MO, USA). Anti-COX2 (PA5-17614), anti-beta actin (BA3R), and HRP-conjugated anti-rabbit (#31460) antibodies were purchased from Thermo Scientific (Rockford, IL, USA), whereas anti-IL-1β (D6D6T) was from Cell Signaling Technology (Danvers, MA, USA).

### 4.2. Plant Material and Extraction

Samples of *Justicia spicigera* were collected in Mexico City, México (21°58′43.90″ N and 98°58′31.44″ W) in October 2019, identified (voucher number FEZA 4656) by a specialist, and preserved at the herbarium School of Higher Studies of Zaragoza, UNAM. Dried leaves of *Justicia spicigera* (40 g) were extracted with 1 L of ethanol using a Soxtherm apparatus (Soxtherm automatic, Gerhadt, Germany) for 3 h. The *Justicia spicigera* extract (JSE) was filtered, evaporated, and then lyophilized (Free Zone 2.5, Labconco, MO, USA). The yield of JSE was 3.4%.

### 4.3. Chemical Standardization of Plant Extract

The quantitation of kaempferitrin, an active compound in JSE, was carried out according to the protocol described by Alonso-Castro et al. [8] using an HPLC method in a Waters 2795 chromatograph (Waters Corp., Milford, MA, USA) instrument.

### 4.4. Animals

Male Wistar rats aged 6–8 weeks (weight range 200–250 g) were from the University of Guanajuato animal facility and were housed in isolated cages at 24 °C under a light-dark cycle of 12:12. The animals were supplied with food and water ad libitum. Each animal was used only once and euthanized at the end of the test by an overdose of anesthetic. This study conducted all the experiments in accordance with the NIH Guide for Treatment and Care for Laboratory Animals [29] and the Official Mexican Norm NOM 062-ZOO-1999, which provide technical specifications for the production, care, and use of laboratory animals. The University of Guanajuato’s ethical committee for research (CIBIUG-P30-2019) revised and approved the protocol of this study. This research also followed the Guidelines on Ethical Standards for Investigations of Experimental Pain in Animals [30].

### 4.5. Antinociceptive Assay

#### 4.5.1. Design

Different groups of rats (n = 6) were used to characterize the dose–response curve of each drug. *Justicia spicigera* extract (JSE) at 30, 100, 150, and 200 mg/kg; naproxen (NPX) at 50, 100, 200, and 300 mg/kg; or tramadol (TML) at 5, 12.5, 25, and 50 mg/kg were orally administered 60 min before the administration with formalin. The drug treatments were dissolved in saline solution as a vehicle. The control group received a saline solution. Once the dose–response curve of each drug was obtained, an experimental effective dose (ED_50_, the dose that produces a response of 50% of the maximum response) value was determined for each compound, and the NPX-JSE and TML-JSE combinations were assessed in the second phase of the formalin test using three different proportions (1:1, 3:1, and 1:3).

#### 4.5.2. Formalin Test

The formalin-induced nociception test was performed as described previously [31]. The rats were placed in transparent plastic cylindrical chambers, positioning a mirror at a 45° angle to prevent any unobstructed view of their paws. The animals were injected into the dorsal surface of the hind paw with 50 µL of dilute formalin (5%) and observed for nociceptive behavior immediately after injection. Nociceptive behavior was quantified as the number of paw flinches during 1 min periods every 5 min up to 60 min after injection. Time courses of antinociceptive response to individual drugs and combinations were constructed by plotting the mean number of paw flinches as a function of time. Dose–response data were quantified as the percentage of reduction in paw flinches relative to the control total in the second phase of the formalin test (15–60 min), where JSE showed antinociceptive effects in the previous study [9]. The percentage of antinociception was calculated according to the following equation:

% Antinociception = ((Control-Drug)/Control) × 100, where “Control” and “Drug” refer to the number of paw flinches elicited by formalin plus saline and formalin plus drug, respectively. At the end of the experiment, an anesthetic overdose euthanized the rats.

### 4.6. Western Blot

The rats from the formalin-induced pain model were euthanized to obtain the right and left hind paws. Approximately 120 mg of tissue were sectioned and placed in a tube containing zirconium beads and 0.6 mL of immunoprecipitation (IP) lysis buffer (Thermo Scientific) supplemented with protease inhibitor. The BeadBug^TM^ system homogenized the tube containing the sample through three cycles of 35 s and 2 min in ice. The homogenate was centrifuged at 16,000× *g* for 10 min at 4 °C, and the supernatant was recovered for measuring the protein content using the Bradford assay. Then, 30 µg of protein per lane was loaded and resolved in 10% SDS-PAGE gel and electro-transferred onto polyvinylidene difluoride (PVDF) membranes, which were blocked with 10% of non-fat dry milk in Tris-buffered saline buffer with 0.1% Tween-20 (TBST) and incubated with the respective primary antibody (1:500 for COX2, 1:200 for IL-1β, and 1:1500 for β-actin) overnight at 4 °C. The membranes were washed with TBST and then incubated with 1:3000 HRP-conjugated anti-rabbit secondary antibody for 1 h at room temperature. The membranes were visualized in x-ray film using an ultra-sensitive enhanced chemiluminescent (ECL) kit (SuperSignal West Femto from Thermo Scientific). The intensity of the bands on the blots was quantified using the ImageJ software version 1.54j [32] and normalized for beta-actin values as a loading control for the densitometry analysis.

### 4.7. Bradford and Myeloperoxidase (MPO) Assays

The quantitation of proteins in the tissue from the rats’ paws in the formalin test and MPO as activity was realized following the same methodology described by Barragan-Galvez et al. [33].

### 4.8. Pharmacokinetic Study

#### 4.8.1. Catheterization of the Femoral Artery

The rats received sodic pentobarbital (50 mg/kg i.p.), and the catheterization was performed in accordance with the protocol of Jespersen et al. [34] with minor modifications. The surgical regions (back of the neck and inner leg) were shaved and disinfected using iodopovidone and 70% ethanol. Each rat was lying prone (on its stomach). Then, a horizontal incision (3 mm) was made on the back of the neck at the level of the shoulder blades with scissors, followed by another incision made in the inguinal area (15 mm) along the natural angle of the hind leg, placing each rat onto its back (supine position). We performed the dissection to separate the connective tissue, and then introduced the catheter into the inguinal area at the back of the neck using a disinfected linear guide of steel. The femoral artery was separated from the nervous and femoral veins. This artery was cannulated for blood sampling with a PE-10 catheter rinsed with heparin in a saline solution (125 U/mL) [34]. The wounds were sutured, and the catheter was secured with a high-tension spring located in the back of the neck region. Each animal was placed in an acrylic cage with free access to water and a standard diet to aid in their recovery from surgery.

#### 4.8.2. Experimental Design

Twelve male Wistar rats were maintained under the same laboratory conditions described above. After 3 days of acclimatization, the rats (308.7 ± 42.25 g) were randomly distributed into two groups (NPX and NPX-JSE). The randomization of groups was performed using the GraphPad QuickCalcs software V5 (San Diego, CA, USA). The animals were catheterized by the femoral artery according to the previously described protocol. Twenty-four hours after the anesthesia and a 12 h fast, the rats received a single oral dose of NPX (30 mg/kg) or NPX (30 mg/kg)-JSE (60 mg/kg). The selection of NPX doses was guided by previous studies [9,26], and this work determined the ED50 of JSE to achieve antinociceptive effects. Five hundred microliter blood samples were obtained at 30, 60, 90, 120, 180, 240, 360, 480, 600, and 1440 min after drug administration [35]. The blood volume drawn was replaced with the same saline solution volume to avoid reducing the circulating volume [35], and the catheter was maintained in sodic heparin (125 U/mL) [34]. Finally, the plasma was obtained by centrifuging blood samples at 11,400 rpm at 4 °C for 10 min (MicroCL 21 Centrifuge, Thermo Fisher, Rockford, IL, USA).

#### 4.8.3. HPLC Analysis

Our laboratory developed a method to determine the quantification of NPX in the rat plasma using reversed-phase high-performance (HPLC). Ten microliters of dicloxacillin (1000 μg/mL) were mixed with 100 µL plasma samples as an internal standard (IS). Afterward, two hundred microliters of acetonitrile were added to eliminate the plasma proteins. This mixture was shaken in a vortex for 1 min and then centrifuged for 5 min at 100,000 rpm (HSIANGTAI, New Taipei City, Taiwan, China). The samples were filtered and analyzed by HPLC at room temperature using a C18 column (4.6 × 250 mm, 5 μm, Agilent, Palo Alto, CA). The mobile phase was a solution of acetonitrile and phosphoric acid (1.46 mM pH 3) 30:70 *v*/*v*. It had a constant flow rate of 1.2 mL/min and a pressure of 2000 ± 24 psi (1500 HPLC pump, Waters, Milford, MA, USA). A UV/Visible Detector (2489 HPLC, Waters, Milford, MA, USA) was used to measure NPX at 225 nm.

### 4.9. Data Analysis and Statistics

The results are shown as the mean ± SEM for 6 animals per group. The dose–response data were analyzed using a one-way analysis of variance followed by Dunnett’s test.

The possible synergism between JSE-NPX and JSE-TML was evaluated by constructing isobolograms using the individual ED_50_ values. The theoretical additive ED_50_ and the experimentally derived ED_50_ values were compared using the Student’s t-test. An experimental ED_50_ value significantly lower than the theoretical additive ED_50_ value indicated a synergistic interaction between JSE-NPX and JSE-TML. The isobolographic analysis was used to characterize drug interactions. The isobologram was constructed by connecting the significantly lower than theoretical additive ED_50_ value of naproxen plotted on the abscissa with the ED_50_ value of JSE plotted on the ordinate to obtain the additive line. For the mixture of JSE and naproxen, the ED_50_ value was determined by the linear regression analysis of the log dose–response curve (six animals with at least four doses) and compared using a t-test to a theoretical additive ED_50_ value [36,37]. The interaction index (γ) was obtained by dividing the ED_50_ value of the experimental combination by the ED_50_ value of the theoretical combination. This index showed the difference between the theoretical and experimental ED_50_ values for each combination. The γ indicates the portion of the ED_50_ value of individual drugs that accounts for the corresponding ED_50_ value in the combination. Values of approximately 1 correspond to an additive interaction; values greater than 1 imply an antagonistic interaction; and values less than 1 indicate a synergistic interaction. Statistical significance was considered when *p* < 0.05.

For the pharmacokinetic study, the NPX plasma concentrations were plotted against time after the drug administration. These curves were constructed using the GraphPad V5 software. The pharmacokinetic parameters of naproxen were determined by a non-compartmental method using the Excel add-on PKSolver [37]. The pharmacokinetics parameters were expressed as the mean ± standard deviation (SD).

## 5. Conclusions

The combination NPX:JSE (3:1) showed antinociceptive effects by decreasing the levels of COX2, IL-1β, and MPO without affecting NPX’s pharmacokinetics.

## Figures and Tables

**Table 1:** Theoretical (Z_add_) and experimental (Z_exp_) ED_50_ values and interaction index for the different ratios for the combinations of NPX:JSE.

Proportion			
	NPX1:JSE1	NPX3:JSE1	NPX1:JSE3
Z_add_ (mg/kg)	173.68 ± 17.49	199.94 ± 13.55	147.42 ± 24.11
Z_exp_ (mg/kg)	107.09 ± 0.01 *	102.44 ± 0.09 *	73.82 ± 0.10 *
Interaction Index	0.617	0.512	0.501

* *p* < 0.05 Z_add_ versus Z_exp_, by Student’s *t*-test. The values were expressed as the mean ± standard error of the mean (SEM).

**Table 2:** Theoretical (Z_add_) and experimental (Z_exp_) ED_50_ values and interaction index for the different ratios for the combinations of TML:JSE.

Proportion			
	TML1:JSE1	TML3:JSE1	TML1:JSE3
Z_add_ (mg/kg)	67.87 ± 15.97	41.22 ± 8.31	94.51 ± 23.85
Z_exp_ (mg/kg)	66 ± 0.02	14.79 ± 0.10 *	39.23 ± 0.03 *
Interaction Index	0.973	0.359	0.415

* *p* < 0.05 Z_add_ versus Z_exp_, by Student’s *t*-test. The values were expressed as the mean ± standard error of the mean (SEM).

**Table 3:** Mean (±standard deviation) pharmacokinetic parameters of naproxen after single oral administration at a dose of 30 mg/kg to the rats with 60 mg/kg of JSE.

Parameter	NPX	NPX + JSE
Tmax (h)	1.25 ± 0.9874	1.16 ± 0.41
t1/2 (h)	17.05 ± 7.46	10.78 ± 8.41
Cmax (ug/mL)	68.70 ± 11.72	55.15 ± 5.86
AUC 0-t (μg/mL^×^h)	650.52 ± 149.01	529 ± 67.54
AUC 0-inf_obs (μg/mL^×^h)	1038.77 ± 208.75	718.65 ± 222.93

## Data Availability

Data are contained in this manuscript.
